# Stress, Health and Well-Being: The Mediating Role of Employee and Organizational Commitment

**DOI:** 10.3390/ijerph10104907

**Published:** 2013-10-11

**Authors:** Ajay K. Jain, Sabir I. Giga, Cary L. Cooper

**Affiliations:** 1 Department of Psychology and Behavioral Sciences, Aarhus University, DK-8000 Aarhus C, Denmark; 2 Division of Health Research, Faculty of Health and Medicine, Lancaster University, Lancaster, LA1 4YW, UK; E-Mail: s.giga@lancaster.ac.uk; 3 Lancaster University Management School, Lancaster University, Lancaster, LA1 4YW, UK; E-Mail: c.cooper1@lancaster.ac.uk

**Keywords:** organizational stress, physical health, psychological well-being, commitment, India, business process outsourcing organizations, ASSET

## Abstract

This study investigates the mediating impact of organizational commitment on the relationship between organizational stressors and employee health and well-being. Data were collected from 401 operator level employees working in business process outsourcing organizations (BPOs) based in New Delhi, India. In this research several dimensions from ASSET, which is an organizational stress screening tool, were used to measure employee perceptions of stressors, their commitment to the organization, their perception of the organization’s commitment to them, and their health and well-being. Data were analyzed using structural equation modeling on AMOS software. Results of the mediation analysis highlight both employee commitment to their organization and their perceptions of the organization’s commitment to them mediate the impact of stressors on physical health and psychological well-being. All indices of the model fit were found to be above standard norms. Implications are discussed with the view to improving standards of health and well-being within the call center industry, which is a sector that has reported higher turnover rates and poor working conditions among its employees internationally.

## 1. Introduction

The purpose of this empirical study within business process outsourcing organizations (BPOs) in India is to examine the mediating role of commitment, both in terms of employee commitment to their organization as well as their perception of their organization’s commitment to them, in the relationship between organizational stressors, physical health and psychological well-being. To examine this research model, we have used ASSET which is an organizational stress screening tool developed in Europe [1,2,3] and also validated and tested in the Indian work context [4]. In this study, ASSET’s sub-scales are tested using structural equation modeling. Moreover, Indian BPOs are a useful research setting to test the validity of this model since the industry has been a focus of many studies relating to the impact of organizational stressors including high work demands, long working hours and permanent night shifts on outcomes such as work-related illness, performance and absenteeism [5,6].

### 1.1. Work and Stress

Since the beginning of the 20th century work stress has been a major topic for researchers and practitioners working in the fields of psychology, organizational behavior, health and medicine [7,8]. Evidence shows that work related stress has a negative impact on employee job performance, and their physical and psychological well-being, including musculoskeletal and immune system complications [9,10]. Stressors can also adversely affect operational efficiency; it is reported to increase employee turnover, accidents and ill health and reduce employee motivation and satisfaction, all of which may impact on the overall functioning and profitability of organizations [11]. Stress is expressed in the form of overwhelming exhaustion, feelings of cynicism and detachment from the job, and a sense of ineffectiveness and lack of accomplishment [12]. Thus there is a plethora of research which confirms the negative effect of organizational stress on employee health and their psychological well-being. However, there is limited attention in the literature on the mediator and moderator effect of commitment on this relationship. Although, stress is an inevitable part of organizational life, effort can be made to reduce its negative effect on health and well-being. Therefore, this research aims to explore the mediating impact of employee and organizational commitment in the relationship between stress and health and well-being within the BPO sector in India.

Stress has been viewed from four main perspectives: as a stimulus [13], as a response [14], as an interaction between a stimulus and response [15] and as a transaction [16]. From a transactional perspective, stress is not a factor that resides in the individual or the environment. Rather, it is embedded in an ongoing process that involves individuals interacting with their environment, making appraisals of those encounters and attempting to cope with issues that arise [8]. Lazarus and Folkman defined stress as “*a particular relationship between the person and the environment that is appraised by the person as taxing or exceeding his or her resources and endangering his or her well-bein*g” ([17], p.19). In this regard an environmental stimuli is not inherently a stressor, rather it becomes one only when individuals perceive it as a threat for them and they think that it is beyond their capacity to deal with [18,19]. In a similar manner, organizational stress is seen as a consequence of a mismatch between job resources and job demands [20].

### 1.2. Dimensions of Organizational Stress

This study aims to test a model fit between three main dimensions of the ASSET questionnaire [1], which are “perceptions of your job”, “attitude towards your organization”, and “your health”. A detailed description of these measures is given below.

#### 1.2.1. Perceptions of Your Job

This scale measures a range of possible sources of workplace stress and job pressure. It also includes some items relating to home and social life-related pressures. This part of the questionnaire comprises of 37 items divided into eight sub-scales, which collectively assess eight sources of stress identified by the ASSET model.

*Work Relationships*: This sub-scale measures the extent to which work relationships are a source of stress. Poor or unsupportive relationships with colleagues and/or superiors, isolation (a perceived lack of adequate relationships) and unfair treatment can all be potential sources of stress.

*Work Life Balance*: Work demands have the potential to spill over and interfere with personal and home lives. ASSET’s work life balance sub-scale measures the extent to which maintaining a satisfactory balance between work responsibilities and personal/home life is a source of stress.

*Overload*: This sub-scale of the ASSET questionnaire measures the extent to which unmanageable workloads and time pressures are a source of stress.

*Job Security*: While significantly fewer employees now expect a “job for life”, the fear of losing one’s job or one’s job becoming obsolete still remains a major potential source of stress. This sub-scale of the questionnaire consequently measures the extent to which insecurity and change are a source of stress.

*Control*: The experience of stress is strongly linked to perceptions of control. Lack of influence over the way in which work is organized and performed can be a potential source of stress. The extent to which a lack of control is perceived by individuals to be a source of stress is addressed by this ASSET sub-scale.

*Resources and Communication*: To perform effectively, individuals need to feel they have the appropriate training, equipment and means available to them. They also need to feel that they are adequately informed and that they are valued. This sub-scale measures the extent to which a lack of resources or communication is perceived by individuals to be a source of stress.

*Pay and Benefits*: The financial rewards that work brings are obviously important in that they contribute to the kind of lifestyle that an individual can potentially lead. In addition, they often influence feelings of self-worth and an individual’s perception of their value to the organization. This single item scale measures the extent to which pay and benefits are a source of stress.

*Aspects of the Job*: This ASSET sub-scale measures potential sources of stress that relate to the fundamental nature of the job itself. It incorporates factors such as physical working conditions, type of tasks and the amount of satisfaction derived from the job itself.

#### 1.2.2. Attitudes towards Your Organization

This section of the questionnaire is concerned with the measurement of commitment. It consists of nine items divided into two scales: commitment of the organization to the employee and commitment of the employee to the organization. This questionnaire reflects the non-economic reciprocal obligations that exist between employer and employee. In terms of relating this section of the questionnaire to the ASSET model, this sub-scale measures an effect of organizational stressors.

*Commitment of the Organization to the Employee*: Employees expect to be trusted and respected and to feel that it is worth them “going the extra mile” for their organization. This sub-scale measures the extent to which individuals feel that their organization is committed to them.

*Commitment of the Employee to the Organization*: Employers expect their employees to do their job as best they can and for them to be loyal and dedicated to the organization. This sub-scale measures the extent to which this commitment exists.

#### 1.2.3. Your Health

This section of the questionnaire assesses respondents’ state of health. It consists of 19 items divided in to two sub-scales: Physical Health and Psychological Well-being. According to the ASSET model and the large body of research on which it is based, poor employee health can be indicative of excessive workplace pressure and the level of stress employee’s experience. Thus, poor health is taken to be an outcome of stress, which can be used to indicate whether workplace pressures have a positive and motivating effect, or conversely, a negative and damaging effect.

*Physical Health*: All items on this sub-scale relate to physical symptoms of stress. The role of this sub-scale is to give an insight into physical health, not an in-depth clinical diagnosis.

*Psychological Well-being*: The items listed on this sub-scale are symptoms of stress-induced psychological ill health. As with the physical health measure, the role of this sub-scale is to give an indication of psychological health, not an in-depth clinical diagnosis.

“Attitude towards your organization” has been used as an outcome variable to stressors in the original ASSET model [1] and also as a moderator variable in other studies [21]. However this paper focuses on the mediating impact of both perceived organizational commitment to employees as well as employee commitment to the organization in the relationship between organizational stressors and employee health and well-being in the of BPO industry in India.

### 1.3. The Proposed Model: Exploring the Mediating Role of Commitment

Stressors may produce a negative impact on health and well-being if employees lack resources to cope with demands. In the psychology literature, attitudinal and dispositional variables are conceptualized as major assets which are available to individuals in the form of self-efficacy, resilience, optimism and hope [22]. Such psychological capacity may mitigate the negative impact of stressors on performance and drive people for higher accomplishments and more challenges [23]. Apart from developing such psychological capacities, organizations are equally willing to foster positive organizational attitudes like job satisfaction and organizational commitment to improve organizational performance [24,25]*.* Evidence shows that stress produces a negative impact on job satisfaction and organizational commitment [26]. Researchers have identified organizational commitment as a significant moderator of stress [27]. Organizational commitment is not only related to many physical and psychological employee outcomes, but also to the moderating effects on the stressor-health relationship. Organizational commitment therefore interacts with sources of stress at work to determine its outcomes. Siu argues that this indirect or moderating effect of commitment protects individuals from the negative impact of stress due to the fact that it enables them to see direction in and attach meaning to their work [27]. Organizational commitment can also provide people with stability and a feeling of belonging. Furthermore, some researchers argue that employee perceptions of their employer’s commitment to them act as a buffer when they are challenged by stressful working conditions [28].

Perceived organizational support is positively related with affective commitment, job satisfaction, performance and citizenship behavior, and negatively related to turnover intentions [29]. Other studies have demonstrated that perceived commitment of the organization to employees moderates the relationship between bullying by superiors and turnover intention [30]. So it can be argued that emotional attachment to the organization may help employees to perform better due to the formation of positive social exchanges [31]. In this way, we can argue that perceived organizational commitment may act as a buffer against organizational demands, and therefore sustain employee physical health and psychological well-being [32,33]. Thus perceived commitment could be related to higher levels of health and well-being and may mediate the relationship between different organizational stressors and indicators of health and well-being. Furthermore, researchers have showed that commitment has a differential impact within individualistic and collectivistic cultural environments [34,35]. For example, there is evidence of the importance of material job value (job quality) in individualistic societies and post-materialistic job values (helping others) in collectivistic societies [35]. Thus, there is a case to explore the mediating impact of commitment on the relationship between stressors and health in the Indian BPO sector. Through encompassing the literature on the relationship between stressors and health we have formulated the following hypotheses:

H1: Organizational stressors will have a negative impact on employee perceptions of their organizations commitment to them as well as the employee’s commitment to the organization.
H2: Organizational stressors will have a negative impact on the physical health and psychological well-being of employees.
H3: Perceived commitment of the organization and employee commitment to the organization will have a negative impact on the physical health and psychological well-being of employees.
H4: Perceived commitment of the organization and employee commitment to the organization will mediate the effect of organizational stressors on the physical health and psychological well-being of employees.

**Figure 1:**
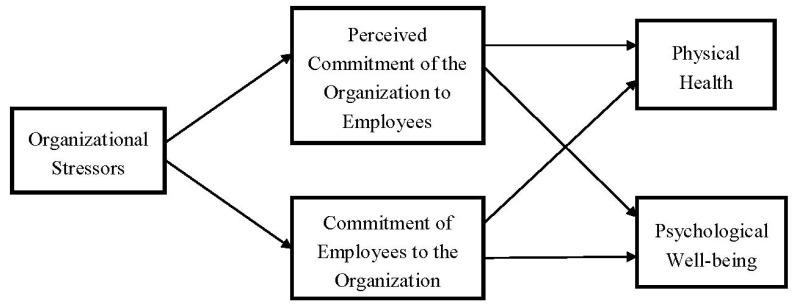
Conceptual scheme of this study.

A mediation effect model was used to carry out this research. The conceptual scheme is presented in Figure 1 below.

## 2. Methods

### 2.1. Participants and Procedure

The sample for this research involves operators from call center organizations located around the national capital of India, including the cities of New Delhi, Noida and Gurgaon. The main role for these operators is to respond to queries originating from customers calling from the USA, UK and other European countries. The call centers provide a round the clock service and operators are required to work different shift patterns often impacting on family life. The work therefore requires employees to work night shifts, be continuously attentive, perform highly repetitive tasks and deal directly with customers—all practices which have been identified as major sources of stress.

Data was collected from 401 operator level employees from five different call centers. The questionnaire was administered with the consent of participants and their HR managers. The sample demographics are as follows: (1) *Age*: a mean age of 24, ranging from 18 to 50 years and with a standard deviation of 3.7 years; (2) *Gender*: 68% male and 32% female; (3) *Education level*: 40% of the respondents had a graduate degree in Arts, Science, Engineering or Commerce. 60% had also completed professional courses in their area of work; (4) *Tenure*: the mean time employees had been in the same organization was 11.44 months with a standard deviation of 9.32 months. Some respondents had only been in their current employment for around one month, whilst others had worked for the organization for 54 months; (5) *Marital Status*: 80% were single, 17% were married, 2% were living with a partner and 1% were divorced.

### 2.2. Measures

A self-report measure based on the ASSET questionnaire [1] was used to collect the data. The primary variables of interest were organizational stressors, employee attitudes towards the organization and employee health.

The organizational stressors measure comprised of 37-items including possible sources of work, home and social stress and consists of 8 factors: *Work Relationships* (WR, α = 0.85), *Your Job* (YJ, α = 0.61), *Overload* (OL, α = 0.81), *Control* (CL, α = 0.75), *Job Security* (JS, α = 0.72), *Resource and Communication* (RC, α = 0.76), *Work-Life Balance* (WLB, α = 0.61) and *Pay and Benefits which* is a single item scale. The first two factors each consist of eight items, the next five factors contain four items and the final factor comprises of a single item. Examples of items are “my relationships with colleagues are poor” (WR evaluates issues arising from contacts people have at work with their colleagues/managers); “my physical working conditions are unpleasant” (YJ relates to the fundamental nature of the job itself); “I do not have enough time to do my job as well as I would like” (OL examines the time pressure and workload); “I am not involved in decisions affecting my job” (CL measures perception of the amount of control over work); “my job skills may become redundant in the near future” (JS measures the level of job security); “I do not have proper equipment or resources to do my job” (RC examines resource availability and effectiveness of communication processes within the organization); “I work longer hours than I would choose to” (WLB evaluates the extent to which demands of work interfere with the respondent’s personal and home life); the last factor “pay and benefits” is a single item measure of the extent to which pay and benefits are considered to be appropriate.

Employee attitude towards the organization comprises of nine items. It has two dimensions, namely perceived commitment of the organization to employees (PCOE, α = 0.86) and perceived commitment of employees to the organization (PCEO, α = 0.73). The first dimension consists of five items and the second dimension has four items. An example of the first dimension is “I feel valued by the organization” and an example of the second dimension is “I am committed to this organization”.

Employee health has 17 items and two dimensions. The first dimension is physical health (PH, α = 0.82), which comprises of six items. The second dimension is psychological well-being (PWB, α = 0.92), which has eleven items. An example of physical health is “lack of appetite or over eating” and an example of psychological well-being is “becoming angry with others too easily”.

Demographic variables such as age, sex, tenure within the organization, education and marital status were used as control variables. All survey items were rated on a 6-point Likert-type scale ranging from 1 (“strongly disagree”) to 6 (“strongly agree”).

### 2.3. Analytic Procedure

As the major aim of this study was to analyze the mediating impact of commitment on the relationship between organizational stress and employee health, data were analyzed using SPSS and AMOS software. Data analysis was carried out in three parts: (1) Confirmatory factor analysis and reliability analysis were used to validate the usefulness of the ASSET sub-scales in this context; (2) Correlational analysis was used to determine the relationship between organizational stressors, perceived commitment and health. Zero-order correlations are measures of direct effect [36], as they determine the magnitude of the bivariate relationship between the independent and dependent variable without accounting for the contributions of other variables; and (3) Mediation analysis was carried out to assess the mediating impact of both commitment measures on the relationship between organizational stress and health and well-being. To test the mediation, the procedures suggested by Baron and Kenny [37] were applied: (a) the independent variable must be related to the mediator; (b) the independent variable must be related to the dependent variable; (c) the mediator must be related to the dependent variable; and (d) the independent variable must have no effect on the dependent variable when the mediator is held constant (full mediation) or should become significantly smaller (partial mediation) [37]. Further analysis was conducted using Sobel’s test in order to determine the strength of the mediating effect of commitment in the relationship between stressors and health [38].

A description of all ASSET subscales is provided in Table 1 below. A comparison of norms from the Indian call centers (BPOs) from this study is made with ASSET [1] norms for the general and managerial/professional population internationally and is available in the Appendices.

**Table 1:** Summary of ASSET sub-scales (*n* = 401).

Scales	Factors/Abbreviations	No. of Items	Cronbach’s Alpha
Perception of your job	1. Work relationships WR	8	0.85
	2. Aspects of your job YJ	8	0.72
	3. Overload OL	4	0.81
	4. Control CL	4	0.75
	5. Job security JS	4	0.72
	6. Resource and communication RC	4	0.76
	7. Work-life balance WLB	4	0.61
	8. Pay and Benefits PB	1	
Attitude towards your organization	Perceived commitment of organization to employee PCOE	5	0.86
	Commitment of employees to organization PCEO	4	0.73
Your health	Physical Health PH	6	0.82
	Psychological Well-Being PWB	11	0.92

## 3. Results and Discussion

### 3.1. Confirmatory Factor Analysis (CFA)

CFA was administered to assess the validity of the ASSET questionnaire. Chi Square, Tucker Lewis Index, Normed Fit Index (NFI), Comparative Fit Index (CFI) and Standardized Root Mean Square Residual (SRMR) are used as indicators of overall model fit to evaluate if the observed covariance matrix fitted the hypothesized model. RMR is a measure of the average discrepancy between fitted and observed covariance matrices. A RMR of less than 0.10 points suggests a good model fit. The CFI compares the relative improvement in fit for a proposed model over a strict null model of complete independence between the various items. Values above 0.90 for CFI suggest an acceptable fit [39]. The CFI is recommended as the best approximation of population value [40]. Table 2 (below) presents the results of the confirmatory factor analysis for all ASSET dimensions. The three-factor model was confirmed. Both a standardized RMR of 0.030 and CFI of 0.97 suggest that the ASSET sub-scales from this sample are valid.

**Table 2:** Results of confirmatory factor analysis for the ASSET questionnaire.

Goodness of Fit Statistics	Model Fit
Minimum Fit Function Chi-Square	75.885 < 0.001
Root Mean Square Error of Approximation (RMSEA)	0.046
Normed Fit Index (NFI)	0.968
Tucker-Lewis fit Index (TLI)	0.975
Comparative Fit Index (CFI)	0.985
Standardized RMR (SRMR)	0.030
Goodness of Fit Index (GFI)	0.970
Adjusted Goodness of Fit Index (AGFI)	0.943

### 3.2. Descriptive Statistics and Correlations

All the descriptive statistics for this study and a correlation matrix are displayed in Table 3 (below). Zero order correlations demonstrate that results are consistent for the first three hypotheses. The data also suggests that all the factors within the organizational stress scale were found to be negatively related with perceived commitment of the organization to employees, commitment of employees to the organization, physical health and psychological well-being. The correlations between factors of ASSET and mediators and criterion variables were consistently negative, which supports the first and second hypotheses. Furthermore, in support of the third hypothesis, the table of correlations below (Table 3) also highlights the positive impact of both the employee’s and organization’s commitment on physical health and well-being.

### 3.3. Mediator Analysis

**Figure 2:**
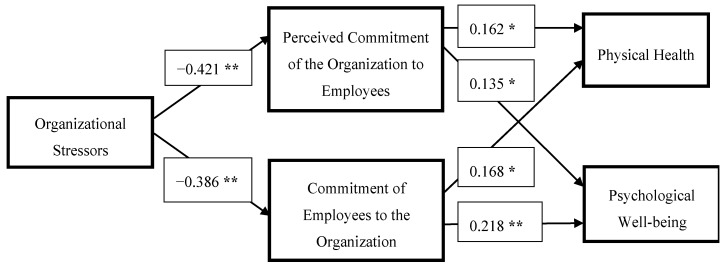
Standardized betas for the mediating impact of commitment (* *p* > 0.05; ** *p* < 0.01).

The major aim of this study was to test the model fit among various sub-scales of ASSET as suggested in the literature. Results of the mediation analysis are presented below in Figure 2 and Table 4.

**Table 3:** Means, standard deviations and correlations for all the variables in this study. Note: ** Correlation is significant at the 0.01 level.

Variables	Mean	SD	1	2	3	4	5	6	7	8	9	10	11
1. Work Relationships	18.47	6.34	1										
2. Work life balence	13.98	4.18	0.432 **	1									
3. Overload	10.67	4.27	0.639 **	0.483 **	1								
4. Job security	12.94	4.16	0.488 **	0.370 **	0.504 **	1							
5. Control	12.01	4.08	0.596 **	0.451 **	0.529 **	0.495 **	1						
6. Resources and communication	10.54	4.19	0.685 **	0.443 **	0.647 **	0.497 **	0.644 **	1					
7. Aspects of the job	23.28	5.67	0.544 **	0.502 **	0.563 **	0.507 **	0.498 **	0.506 **	1				
8. Pay and benefits	3.67	1.59	0.336 **	0.334 **	0.381 **	0.386 **	0.337 **	0.405 **	0.362 **	1			
9. Commitment of organization	9.65	2.10	−0.372 **	−0.293 **	−0.344 **	−0.331 **	−0.339 **	−0.390 **	−0.261 **	−0.232 **	1		
10. Commitment of employees	9.4	1.81	−0.367 **	−0.257 **	−0.350 **	−0.282 **	−0.331 **	−0.337 **	−0.213 **	−0.214 **	0.724 **	1	
11. Physical health	14.99	4.44	−0.336 **	−0.457 **	−0.432 **	−0.277 **	−0.344 **	−0.369 **	−0.375 **	−0.184 **	0.239 **	0.225 **	1
12. Psychological well being	32.73	7.72	−0.446 **	−0.388 **	−0.473 **	−0.408 **	−0.379 **	−0.403 **	−0.385 **	−0.193 **	0.334 **	0.319 **	0.673 **

**Table 4:** Results of structural equation modeling testing the role of perceived commitment as a mediator in the relationship between organizational stressors and health.

Goodness of Fit Statistics	Model Fit
Chi-Square	8.64 < 0.013
Root Mean Square Error of Approximation (RMSEA)	0.081
Normed Fit Index (NFI)	0.986
Relative Fit Index (RFI)	0.931
Incremental Fit Index (IFI)	0.989
Tucker-Lewis Index	0.946
Comparative Fit Index (CFI)	0.989
Standardized RMR	0.043
Goodness of Fit Index (GFI)	0.992
Adjusted Goodness of Fit Index (AGFI)	0.936

Results of mediator analysis (Table 4 above) suggest that both perceived commitment of the organization to employees and employee commitment to the organization mediate in the relationship between organizational stressors and physical health and psychological well-being. All the model fit statistics are above the standard values [41]. Results of Sobel’s tests support the significant mediation impact of perceived commitment of the organization to employees and perceived commitment of employees to the organization. Sobel’s test statistics for the mediating effect of perceived commitment of the organization to employees were −2.18 (<0.01) (physical health) and −2.99 (*p* < 0.01) (psychological well-being) and for the mediating effect of perceived commitment of employees to the organization were −1.68 (*p* < 0.05) (physical health) and −2.28 (*p* < 0.01) (psychological well-being). Thus the results from Sobel’s test suggest that perceived commitment has significantly carried the effect of organizational stressors on physical health and psychological well-being. However, perceived commitment of the organization had a stronger impact on health and well-being in this context. These results therefore support the notion that organizational support may have a direct positive effect on health and well-being and can also protect against the negative effects of stressors [32,33].

## 4. Limitations and Conclusions

This study investigates the mediating impact of commitment, both in terms of employee commitment to their organization as well as their perceptions of the organizations commitment to them, on the relationship between organizational stressors, and the physical health and psychological well-being of operators working in BPOs in India. All the variables were adopted from ASSET, which is a widely used stress audit tool.

The limitations of this study include its dependence on self-reported measures and its cross-sectional design. The cultural and sector specific context of this study may also limit the generalisability of its findings. Future research may further explore the role of cross cultural issues on commitment, particularly in other professional settings such as in education and healthcare as well as in a more individualistic environment [35]. Nonetheless, this research has identified that organizational stressors have a negative impact on employee perceptions of commitment and their health and well-being, and commitment significantly mediates the impact of stressors on negative outcome variables. Through the use of a SEM approach, the results of this study highlight the usefulness of ASSET within a BPO sector environment and demonstrate how attitude towards the organization has a significant mediating impact. Attitude to the organization is generally observed as an outcome variable, including in the original ASSET model [1] and as a moderator variable in some other studies [21]. This study uses attitude to the organization as a mediator variable in the context of the BPO sector in India. This sector experiences higher than average levels of employee turnover and other work related issues. This research supports the argument that employer and employee commitment can be seen as a moderator or mediator [27,30,31]. However, the analysis highlights a stronger emphasis on the role of perceived organizational commitment to employees in comparison to employee commitment to the organization [28]. This further validates the role of social support in promoting positive adjustment (the “main-effect” model) and in protecting against the negative effects of stress (the “buffering” model) [32,33].

These findings aim to broaden the view on the role of employee commitment as used in the ASSET questionnaire. Although stressors may produce a negative effect on employee commitment and their health, commitment acts as a powerful mediator in regulating the negative effect of stressors on employee well-being. Since commitment is not only influenced by stressors at work but also by other intra-individual and extra-individual factors such as self-efficacy, age, education, tenure, organizational support, fairness and justice, employees’ positive emotional reactions may help in reducing the negative impact of stressors. This is supported by the literature, which highlights the moderating role of emotional intelligence in controlling the negative impact of work stressors [42]. The results of this study suggest that employee commitment to their organization and their perceptions of employer commitment to them have controlled the negative impact of organizational stressors on their health and well-being. From a positive psychology perspective, a focus on developing more optimistic attitudes in organizational contexts can enhance psychological capital [22] as well as play a motivational role [43] in mitigating the effects of stressors on physical health and psychological well-being.

## Appendix. Current Indian BPO Sample (*n* = 401) Comparisons with ASSET Norms

### A1. Perception of Your Job

#### A1.1. Work Relationships

**Table:**

Item Number	General Population Norms (*n* = 25,352)		Managerial and Professional Norms (*n* = 5,947)		Indian Call Center (*n* = 401)	
9	2.44	0.66	2.18	1.44	2.05	1.23
11	3.14	0.68	3.09	1.56	2.38	1.24
18	2.94	0.56	2.56	1.39	2.47	1.25
19	2.69	0.33	2.56	1.36	2.42	1.30
20	3.36	0.33	3.47	1.59	2.82	1.34
23	2.79	1.04	1.97	1.10	2.78	1.14
24	2.37	0.38	2.59	1.37	2.43	1.21
26	2.57	0.92	1.83	0.92	1.78	1.02
Total Score	21.85	2.85	20.26	7.13		

#### A1.2. Work-Life Balance

**Table:**

Item Number	General Population Norms (*n* = 25,352)		Managerial and Professional Norms (*n* = 5,947)		Indian Call Center (*n* = 401)	
1	3.34	0.33	3.59	1.69	3.63	1.52
2	3.13	0.65	3.31	1.87	3.59	1.64
3	2.45	0.37	2.30	1.39	3.54	1.76
5	3.53	0.37	3.67	1.61	3.25	1.58
Total Score	12.43	1.24	12.87	4.60		

#### A1.3. Overload

**Table:**

Item Number	General Population Norms (*n* = 25,352)		Managerial and Professional Norms (*n* = 5,947)		Indian Call Center (*n* = 401)	
15	2.59	0.43	2.52	1.34	2.59	1.41
21	2.58	0.40	2.82	1.39	2.84	1.64
22	2.92	0.27	3.19	1.52	2.70	1.76
32	3.24	0.69	4.06	1.62	2.58	1.58
Total Score	11.33	1.27	12.59	4.55		

#### A1.4. Job Security

**Table:**

Item Number	General Population Norms (*n* = 25,352)		Managerial and Professional Norms (*n* = 5,947)		Indian Call Center (*n* = 401)	
12	3.00	0.62	2.44	1.48	3.13	1.53
13	2.38	0.35	2.14	1.58	3.24	1.54
33	3.58	0.56	4.09	1.44	3.51	1.43
34	2.70	0.34	2.37	1.28	3.02	1.37
Total Score	11.66	0.81	11.04	3.87		

#### A1.5. Control

**Table:**

Item Number	General Population Norms (*n* = 25,352)		Managerial and Professional Norms (*n* = 5,947)		Indian Call Center (*n* = 401)	
4	3.68	0.39	3.67	1.53	3.39	1.53
29	3.17	0.46	3.59	1.54	3.19	1.49
35	2.90	0.44	2.99	1.41	2.82	1.36
36	3.27	0.48	3.04	1.46	2.65	1.34
Total Score	13.02	0.98	13.3	4.73		

#### A1.6. Resources and Communication

**Table:**

Item Number	General Population Norms (*n* = 25,352)		Managerial and Professional Norms (*n* = 5,947)		Indian Call Center (*n* = 401)	
27	3.53	0.42	3.57	1.56	2.84	1.54
28	3.32	0.56	3.72	1.61	2.77	1.42
30	2.94	0.42	2.78	1.43	2.60	1.39
31	3.02	0.54	3.05	1.52	2.35	1.26
Total Score	12.82	0.94	13.12	4.40		

#### A1.7. Aspects of the Job

**Table:**

Item Number	General Population Norms (*n* = 25,352)		Managerial and Professional Norms (*n* = 5,947)		Indian Call Center (*n* = 401)	
6	3.75	0.45	3.99	1.67	2.83	1.70
7	2.93	0.53	3.05	1.69	2.04	1.32
8	3.04	0.94	2.83	1.71	1.76	1.13
10	3.14	0.68	3.28	1.44	4.58	1.40
16	3.06	0.59	3.23	1.58	3.10	1.47
17	2.60	0.44	2.25	1.24	3.22	1.52
25	3.64	0.45	3.80	1.59	3.32	1.52
37	3.29	1.02	2.34	1.35	2.50	1.40
Total Score	25.46	2.77	24.76	6.64		

#### A1.8. Pay and Benefits

**Table:**

Item Number	General Population Norms (*n* = 25,352)		Managerial and Professional Norms (*n* = 5,947)		Indian Call Center (*n* = 401)	
14	3.44	0.33	3.31	1.75	3.67	1.61

### A2. Attitude towards Your Organization

#### A2.1. Employee Perceptions of Their Organizations Commitment to Them

**Table:**

Item Number	General Population Norms (*n* = 25,352)		Managerial and Professional Norms (*n* = 5,947)		Indian Call Center (*n* = 404)	
1	3.94	0.53	3.49	1.53	4.29	1.29
4	3.99	0.40	4.18	1.48	4.41	1.39
5	4.18	0.22	3.89	1.38	4.71	1.24
6	3.98	0.47	3.62	1.45	4.35	1.28
7	4.03	0.29	3.93	1.41	4.46	1.25
Total Score	20.11	1.24	19.12	5.34		

#### A2.2. Employee Commitment to Their Organization

**Table:**

Item Number	General Population Norms (*n* = 25,352)		Managerial and Professional Norms (*n* = 5,947)		Indian Call Center (*n* = 401)	
2	4.36	0.34	4.42	1.34	4.40	1.20
3	4.34	0.41	4.35	1.34	4.84	1.05
8	3.41	0.94	4.23	1.34	4.46	1.29
9	3.47	1.11	4.08	1.37	4.83	1.15
Total Score	15.58	2.53	17.08	3.92		

### A3. Your Health

#### A3.1. Physical Health

**Table:**

Item Number	General Population Norms (*n* = 25,352)		Managerial and Professional Norms (*n* = 5,947)		Indian Call Center (*n* = 401)	
1	2.42	0.26	2.15	1.02	2.66	1.00
2	2.33	0.31	2.06	1.04	2.58	1.06
3	2.21	0.44	2.50	1.04	2.62	1.09
4	2.59	0.21	2.43	0.99	2.55	1.03
6	2.44	0.21	2.55	1.04	2.43	1.07
7	1.83	0.27	1.62	0.82	2.22	1.02
Total Score	13.82	0.77	13.32	4.08		

#### A3.2. Psychological Well-Being

**Table:**

Item Number	General Population Norms (*n* = 25,352)		Managerial and Professional Norms (*n* = 5,947)		Indian Call Center (*n* = 401)	
5	1.68	0.16	1.63	0.87	1.89	1.04
8	2.15	0.14	2.21	0.91	2.06	0.98
9	2.16	0.26	2.09	0.87	1.92	0.90
10	2.37	0.48	2.12	0.89	1.84	0.99
11	2.25	0.16	2.35	0.89	2.00	1.02
12	2.43	0.46	2.83	0.98	2.37	1.02
13	2.09	0.17	2.14	0.90	1.95	0.91
14	1.86	0.16	1.91	0.90	1.94	1.06
15	2.00	0.19	2.03	0.89	2.32	1.00
16	1.86	0.14	1.83	0.80	1.87	0.93
17	2.00	0.33	2.27	0.87	2.06	0.95
Total Score	23.15	1.38	23.07	2.30		
